# Choosing the right reference cohort for assessing outcome of venovenous ECMO

**DOI:** 10.1186/s13054-021-03880-3

**Published:** 2022-01-10

**Authors:** Alexander Supady, Paul M. Biever, Dawid L. Staudacher, Tobias Wengenmayer

**Affiliations:** 1 Department of Medicine III (Interdisciplinary Medical Intensive Care), Medical Center – University of Freiburg, Faculty of Medicine, University of Freiburg, Hugstetter Strasse 55, 79106 Freiburg, Germany; 2 Department of Cardiology and Angiology I, Heart Center, University of Freiburg, Freiburg, Germany; 3 Heidelberg Institute of Global Health, University of Heidelberg, Heidelberg, Germany

**Keywords:** COVID-19, Extracorporeal membrane oxygenation, Patient selection

We thank Karagiannidis et al. for reporting important mortality data of COVID-19 patients supported with extracorporeal membrane oxygenation (ECMO) in Germany [1]. The mortality they reported was notably higher than in other large cohorts [2]. The data come from nationwide billing data of all hospitals in Germany; therefore, the risk of selection bias or underreporting of negative results is lower than in previous cohorts.

The authors argue against the unselective use of ECMO in patients who have a high risk of dying during the course of treatment. We agree that ECMO is an expensive and resource-intensive support option. Therefore, it should be used only in selected patients after careful risk–benefit evaluation. This assessment should take into account all available and relevant prognostic information and the (presumed) patient will. During the COVID-19 pandemic, the availability of trained medical staff and the utilization of available intensive care resources must also be taken into consideration [3].

Yet, one of the major difficulties in patient selection is to predict with sufficient accuracy the prognosis of individual patients. This is true for both patients with ECMO support and without. A more liberal use of ECMO in healthier patients who might have had a favorable outcome even without ECMO improves the survival probability attributed to the procedure, as does a restrained use in very sick and older patients who have a rather poor prognosis with or without ECMO (Fig. 1) [2, 4]. However, in our view, the expansion of the scope of indications may nevertheless be justified or even warranted. This may be the case when experienced physicians base their decisions on a responsible bedside assessment of the individual patient and expect poorer outcome without the use of ECMO. This approach was recently described as “salvage ECMO” [5].

**Fig. 1:**
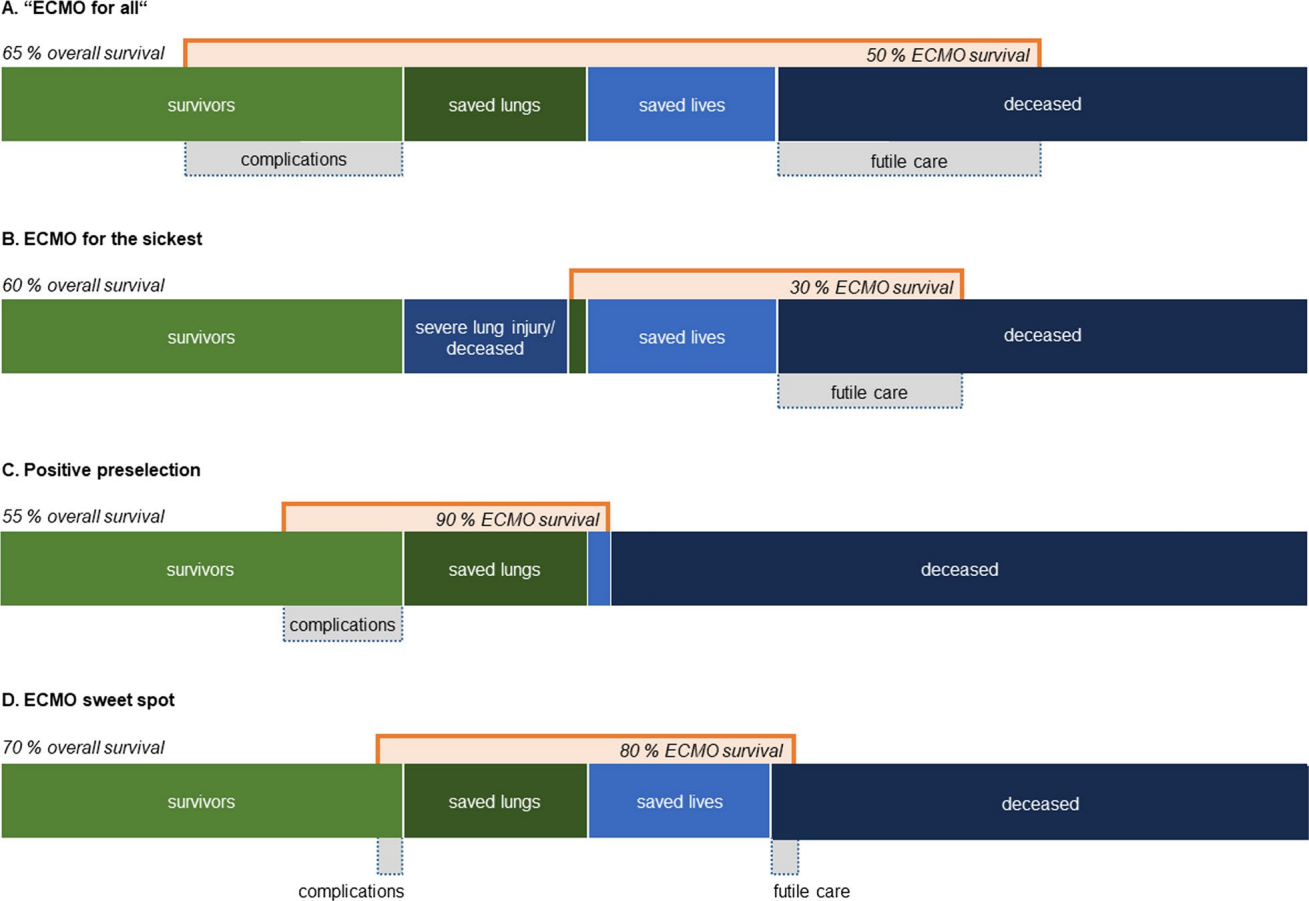
Graphical representation of the challenges of adequate patient selection for ECMO. A very liberal provision of ECMO (“ECMO for all”), considering ECMO for a large proportion of patients, both very severely and less severely diseased (**A**), will achieve the highest absolute number of ECMO survivors. This will be at the expense of a comparably high number of complications (fatal and non-fatal) in patients who would have survived even without ECMO and a large number of futile treatments. When ECMO is considered only for the sickest patients, the proportion of futile care is high and the overall ECMO survival is low; ECMO-related complications in healthier patients may be prevented at the expense of serious irreversible ventilator-induced lung injury, leading to invalidity or death (**B**). Positive preselection and provision of ECMO only for those patients that are having a high chance of survival will ensure prevention of irreversible lung injury, but at the expense of ECMO-related complications in a number of patients who would have survived even without ECMO. Overall survival of the entire cohort treated with ECMO and without is lowest, as compared to the other approaches (**C**). We argue for a strategy of rational ECMO use aiming at the optimal balance between “saved lungs” and limited complications on the one hand and “saved lives” and limited futile care on the other hand (**D**). This approach is likely to achieve the highest number of patients surviving in the entire cohort. *ECMO* extracorporeal membrane oxygenation

For a comprehensive appraisal of the role of ECMO for the treatment of severe respiratory failure, we believe that it is not sufficient to look at the survival rates of patients who eventually received ECMO. Instead, the entire cohort of invasively ventilated patients with defined severity criteria must be considered, including patients in whom ECMO was not chosen, both due to futility and based on the assessment that they will recover even without ECMO. Consequently, the approach that achieves the best overall outcome should be considered superior. This way may help us to offer each patient the optimal therapy and improve our algorithms for making decisions for or against the use of ECMO.

## Data Availability

Not applicable.

## References

[1] Karagiannidis C, Slutsky AS, Bein T, Windisch W, Weber-Carstens S, Brodie D (2021). Complete countrywide mortality in COVID patients receiving ECMO in Germany throughout the first three waves of the pandemic. Crit Care.

[2] Barbaro RP, MacLaren G, Boonstra PS, Combes A, Agerstrand C, Annich G, Diaz R, Fan E, Hryniewicz K, Lorusso R (2021). Extracorporeal membrane oxygenation for COVID-19: evolving outcomes from the international Extracorporeal Life Support Organization Registry. Lancet.

[3] Supady A, Badulak J, Evans L, Curtis JR, Brodie D (2021). Should we ration extracorporeal membrane oxygenation during the COVID-19 pandemic?. Lancet Respir Med.

[4] Supady A, Taccone FS, Lepper PM, Ziegeler S, Staudacher DL (2021). Group CO-S: survival after extracorporeal membrane oxygenation in severe COVID-19 ARDS: results from an international multicenter registry. Crit Care.

[5] Abrams D, Ferguson ND, Brochard L, Fan E, Mercat A, Combes A, Pellegrino V, Schmidt M, Slutsky AS, Brodie D (2019). ECMO for ARDS: from salvage to standard of care?. Lancet Respir Med.

[6] MacLaren G, Fisher D, Brodie D (2022). Treating the Most Critically Ill Patients With COVID-19: The Evolving Role of Extracorporeal Membrane Oxygenation. JAMA..

[7] Beauchamp T, Childress J (2001). Principles of biomedical ethics.

[8] Bercker S, Petroff D, Polze N, Karagianidis C, Bein T, Laudi S, Stehr SN, Voelker MT (2021). ECMO use in Germany: an analysis of 29,929 ECMO runs. PLoS ONE.

