# Supplementary material for: Choosing the right reference cohort for assessing outcome of venovenous ECMO
Source: Crit Care. 2022 Jan 10;26:17. doi: 10.1186/s13054-021-03880-3 (PMC8743758; doi:10.1186/s13054-021-03880-3)
Supplement: Related texts

# Reply: Response to Supady et al.

**Authors:** Thomas Bein, Christian Karagiannidis, Steffen Weber-Carstens, Arthur Slutsky, Wolfram Windisch, Daniel Brodie

**Affiliations:** 4 grid.7727.5 0000 0001 2190 5763 Faculty of Medicine, University of Regensburg, Regensburg, Germany; 5 Department of Pneumology and Critical Care Medicine, ARDS and ECMO Centre, Cologne-Merheim Hospital, Kliniken Der Stadt Köln gGmbH, Witten, Germany; 6 grid.411097.a 0000 0000 8852 305X Herdecke University Hospital, Ostmerheimer Strasse 200, 51109 Cologne, Germany; 7 grid.6363.0 0000 0001 2218 4662 Department of Anesthesiology and Operative Intensive Care Medicine (CCM, CVK), Charité - Universitätsmedizin Berlin, Berlin, Germany; 8 grid.17063.33 0000 0001 2157 2938 Keenan Research Centre for Biomedical Science, Li Ka Shing Knowledge Institute, St Michael’s Hospital, University of Toronto, Toronto, Canada; 9 grid.21729.3f 0000000419368729 Department of Medicine, Columbia University College of Physicians and Surgeons, New York, USA; 10 grid.413734.6 0000 0000 8499 1112 Center for Acute Respiratory Failure, New York-Presbyterian Hospital, New York, USA

We thank Dr. Supady and colleagues for their thoughtful letter regarding the difficulty with divining who should be placed on ECMO based on our data demonstrating high mortality with the use of ECMO for COVID-19 in Germany [1]. Implicit in their argument is that declaring a mortality rate to be high or low requires that we know what mortality rate should actually be expected without the use of ECMO in COVID-19, an information that uncontrolled observational data cannot provide [6] . This is an important argument for conducting well-designed randomized clinical trials. However, absent such evidence, how should we proceed? Nonetheless, we believe that the overall mortality rate should be seen as high relative to the investment of resources, suggesting that the use of ECMO in this population may indeed be too liberal.

One further point that merits discussion is that, while Supady et al. use the term “selection” regarding the decision to implement ECMO, we prefer the term *“*indication.” Indication is based on the careful assessment of the realistic rehabilitation potential of the patient in association with a given intervention (e.g., ECMO) to allow the patient to resume a reasonable quality of life and to achieve a well-defined therapeutic goal in concert with the goals and values of the individual patient [7]. This concept also aims to balance benefit (survival) against harm (invasive treatment, pain, sedation, immobilization and the psychological burden on surrogates). Therefore, the indication for ECMO should be based on several anamnestic, demographic, medical and prognostic parameters in each individual patient to avoid futile treatments as well as high in-hospital mortality. We agree with Dr. Supady’s framing of the balanced and rational use of ECMO; however, the reality in Germany—as expressed in our letter—shows that we are far from the rational use of ECMO. This has also been clearly emphasized by a recent paper showing that 40% of ECMO cases in Germany are performed in hospitals with 2 or fewer cases per year [8] with no assurances regarding the quality of ECMO delivery, which may reasonably be expected to contribute to high mortality.

For these reasons, we are confident that the high mortality rate in German ECMO patients reflects an opportunity to insist on a more rational ECMO application, to reflect both on the organizational approach to its use and on the indications that should be broadly acceptable.
